# Development of a Resveratrol Nanoformulation for the Treatment of Diabetic Retinopathy

**DOI:** 10.3390/ma17061420

**Published:** 2024-03-20

**Authors:** Juliana Gonzalez-Perez, A. M. Lopera-Echavarría, Said Arevalo-Alquichire, Pedronel Araque-Marín, Martha E. Londoño

**Affiliations:** 1 Grupo de Investigación en Ingeniería Biomédica (GIBEC), Universidad EIA, Envigado 055428, Colombia; juliana.gonzalez@eia.edu.co (J.G.-P.); aura.lopera@eia.edu.co (A.M.L.-E.); 2 Schepens Eye Research Institute of Massachusetts Eye and Ear, Boston, MA 02114, USA; sarevalo-alquichire@meei.harvard.edu; 3 Department of Ophthalmology, Harvard Medical School, Boston, MA 02114, USA; 4 Grupo de Investigación en Ciencias Médicas, Universidad EIA, Envigado 055428, Colombia; pedronel.araque@eia.edu.co

**Keywords:** diabetic retinopathy, nanosuspension, resveratrol, cell viability, cell proliferation, cell migration

## Abstract

Diabetic retinopathy (RD) is a microvascular disease that can cause the formation of fragile neovessels, increasing the risk of hemorrhages and leading to vision loss. Current therapies are based on the intravitreal injection of anti-VEGF (vascular endothelial growth factor), which is invasive and can cause secondary effects. The development of new treatments that complement the current therapies is necessary to improve the patient’s outcomes. Nanostructured formulations offer several advantages regarding drug delivery and penetration. In this research, a resveratrol nanosuspension (RSV-NS) was prepared and characterized using dynamic light scattering, field emission scanning electron microscopy, and infrared spectroscopy. The RSV-NS had an average particle size of 304.0 ± 81.21 nm with a PDI of 0.225 ± 0.036, and a spherical-like morphology and uniform particle distribution. Cell viability, proliferation, and migration were tested on endothelial cells (HMRECs). RSV-NS in a concentration of less than 18.75 µM did not have a cytotoxic effect on HMRECs. Likewise, proliferation and migration were significantly reduced compared to the unstimulated control at 37.5 µM. The RSV-NS did not present cytotoxic effects but decreased cell proliferation and migration, indicating that it could provide an important contribution to future medical implementations and could have a high potential to treat this disease.

## 1. Introduction

Diabetic retinopathy (DR) is a complication of diabetes mellitus where the retina’s blood vessels are affected. It is characterized by retinal ischemia, microaneurysms, hemorrhages, poplar spots, intraretinal microvascular abnormalities, venous caliber abnormalities, neovascularization, increased retinal vascular permeability, and blindness [1]. The therapies used to treat DR are intravitreal injections, focal laser treatment, vitrectomy, anti-VEGF (vascular endothelial growth factor) agents, corticosteroids, and antiangiogenic therapy. Although they can reduce the risk of vision loss, they do not entirely attenuate the clinical progression of the disease. They are highly invasive and painful, generating serious secondary effects after application [2]. For this reason, there is a need to create a novel minimally invasive therapy.

**Figure 1:**
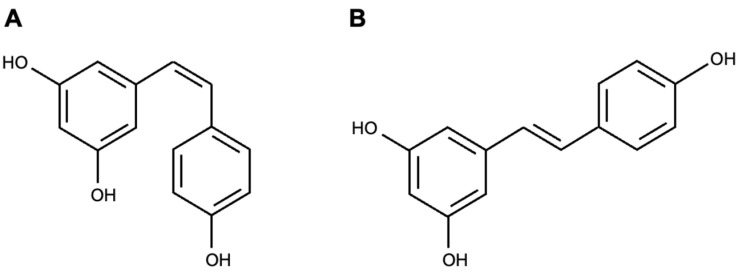
Chemical structures of (**A**) *cis*-resveratrol and (**B**) *trans*-resveratrol isomers.

There are different therapeutic agents or active ingredients that have been described for the treatment of ocular neovascularization, including curcumin [3], quercetin [4], vitamin D [5], and resveratrol [6], among others. Resveratrol (RSV) is a natural, non-flavonoid polyphenol in some plants and fruits, such as peanuts, blackberries, blueberries, grapes, and red wine [7]. It has antioxidant, anti-inflammatory, and anti-allergenic properties, and it can inhibit neovascularization [7,8,9]. RSV consists of two phenolic rings linked by an ethylene bridge that can exist in both *trans* and *cis* forms, as shown in Figure 1, but it is known that the *trans* form is more stable and bioactive than the *cis* form [10,11]. However, its low solubility and bioavailability in target tissues are limited, so current studies have developed formulations to overcome these limitations and provide adequate therapeutic amounts of RSV at the site of action [12,13,14,15].

Carboxymethylcellulose (CMC) is a water-soluble anionic polymer composed of linear molecular chains obtained from the union between cellulose and monochloroacetic acid [16]. CMC has hydrophilic characteristics, it is sensitive to pH or external stimuli, bioadhesion, and controlled release, and is a non-cytotoxic polymer [17]. It is also a promising drug carrier due to its ability to absorb fluids and release them in a controlled manner [18].

One of the decisive challenges when facing diseases in the posterior segment of the eye is the inefficiency in the systemic delivery of therapeutic agents since there are barriers that limit the administration of drugs at non-harmful concentrations [19]. In this context, the search for new drug delivery systems for ocular drug delivery has generated considerable attention, and it has become an important research field in nanotechnology [20]. The implementation of nanocarriers in retinal diseases is considered a promising approach, given that they can improve the bioavailability, increase the permeability, and reduce the degradation of the unstable drugs in the anterior and posterior segments of the eye, minimizing the frequency of invasive therapies and their adverse side effects [21]. 

Several types of nanocarriers have been investigated in ocular drug delivery, such as nanoemulsions, nanosuspensions, nanomicelles, liposomes, dendrimers, solid lipid nanoparticles, polymeric nanoparticles, and others [21,22,23]. Nanosuspensions (NSs) have generated much interest in this field since they allow membrane permeability and improve drug solubility, generating greater penetration into the ocular structure, which facilitates the absorption of the active ingredient [24,25,26].

RSV nanosuspensions have been studied in oral [27], dermal [28], brain [29], and lung applications [30], wherein the dissolution rate, bioavailability, and saturation of the nanosuspensions were significantly higher than those of free RSV [27,29]. Hao et al. fabricated a gelling formulation containing RSV nanoparticles for intranasal drug delivery to the brain, improving mucous membrane permeability and preventing runoff from the nasal cavity. In this formulation, an RSV nanosuspension was prepared with a liquid antisolvent precipitation technique, followed by an in-situ gelling polymer, and, finally, both were mixed. The composite nanosuspension was delivered via intranasal and intravenous administrations for pharmacokinetic and brain-targeting efficiency analyses, respectively [29]. The saturation solubility and dissolution rate of the nanosuspension were significantly enhanced as compared with free RSV, conserving its antioxidant activity [31]. Furthermore, the bioavailability in the brain was increased intranasally with the formulation, and drug targeting efficiency demonstrated a direct delivery through the nose–brain pathway [29].

To the best of our knowledge, no research has been reported using RSV as an active ingredient in a nanosuspension as a potential eye drop treatment for DR. This research aimed to obtain and characterize, physicochemical and biologically, a nanosuspension composed of CMC and RSV (RSV-NS) for the possible application of treating DR.

## 2. Materials and Methods

### 2.1. Materials

CMC (MW ~250 kDa; DS 0.7), RSV, Tween 20 (T20), Span 20 (S20), ethanol (EtOH), and dimethyl sulfoxide (DMSO) were purchased from Sigma-Aldrich Corp. (St. Louis, MO, USA) and glycerin (GLC) from Merck KGaA (Darmstadt, Germany). Ultrapure water was used for the CMC solution using a Barnstead MicroPure purification system from Thermo Fisher Scientific Inc. (Waltham, MA, USA). All chemicals and solvents were analytical grades. Human microvascular retinal endothelial cells (HMRECs) were purchased from Cell Systems Corporation (Kirkland, WA, USA). Endothelial basal media (EBM-2), L-glutamine, and a supplement kit (SingleQuots) were purchased from Lonza (Basel, Switzerland) and fetal bovine serum (FBS) from Atlanta Biologicals (Oakwood, GA, USA).

### 2.2. RSV-NS Preparation

Initially, RSV was dissolved in EtOH at 40 mg/mL. Then, 16 mg of T20, 4.16 mg of S20, and 4 mg of GLC were added successively and stirred vigorously. This solution was called a master mix (MM). Next, the MM was dripped manually with a 100 U insulin syringe and a 28G × 1/2″ needle into 40 mL of a 0.1% CMC solution at room temperature, while being stirred with a D100 hand-held homogenizer from Benchmark Scientific (Sayreville, NJ, USA) at 20,000 rpm. This experiment was performed in triplicate, both for the vehicle (VEH), which did not contain the drug, and for RSV-NS. The concentration of RSV used in the preparation of the RSV-NS (438 µM) was assumed to be the final concentration for the physicochemical characterization.

### 2.3. RVS-NS Physicochemical Characterization

The particle size and polydispersity index of the RSV-NS were determined using the dynamic light-scattering (DLS) technique on a nanoPartica SZ-100V2 de HORIBA Scientific (Piscataway, NJ, USA). The samples were evaluated at room temperature (25 °C). The morphology of the RSV-NS was analyzed by scanning transmission electron microscopy (STEM) using a Thermo Fisher Scientific Inc. (Waltham, MA, USA) Apreo 2 S microscope with an acceleration voltage of 30 kV, high vacuum, and magnification of 150,000–350,000×. RSV-NS and controls were analyzed with Spectrum 100 Series from PerkinElmer (Waltham, MA, USA) using a range from 4000 to 500 cm^−1^. 

### 2.4. RSV-NS Biological Characterization

HMRECs were purchased at passage 3 (P3) and expanded until P7 using endothelial basal media supplemented with 2% FBS, 0.2% gentamicin, 1% L-glutamine, and SingleQuots, and incubated at 37 °C with 5% CO_2_. Cells at P7 were treated in endothelial basal media supplemented with 2% FBS, 0.2% gentamicin, 1% L-glutamine, and selected stimulants. Cell starvation medium was only EBM-2. Previously, plates were coated with a 0.2% gelatin solution to improve cell attachment.

#### 2.4.1. Cell Cytotoxicity Assay

To determine the possible cytotoxic effect of the obtained formulation, toxicity tests were carried out by direct contact on HMRECs by the metabolic reduction method of 3-(4,5-dimethylthiazol-2-yl)-2,5-diphenyltetrazole (MTT) bromide and a Cell Counting Kit (CCK-8) from Sigma-Aldrich Corp. (St. Louis, MO, USA). Briefly, cells were cultured at 5000 cells/well in 96-well plates for 24 h at 37 °C and 5% CO_2_. After the incubation period, the culture medium was removed, and subsequently, the RSV-NS was added at different concentrations of RSV for 24 h.

For the MTT assay, the culture medium was discarded after incubation; a 10% MTT solution was added and incubated for 4 h and protected from light to allow the formation of formazan crystals. Then, the supernatant was removed and DMSO was added to dissolve the crystals. The optical density was measured in a Multiskan Go spectrophotometer of Thermo Fisher Scientific Inc. (Waltham, MA, USA) at 570 nm. For the CCK-8 assay, 10% of the CCK-8 solution was added to the plate, incubated for 2 h, and protected from light. The optical density was read in a spectrophotometer at 450 nm. 

#### 2.4.2. Cell Migration Assay

Migration was evaluated using the scratch wound test reported by Whitmore [32]. Initially, cells were treated with the RSV-NS with different concentrations of RSV for 24 h. Then, the treatment medium was changed to an EBM-2 starvation medium for 12 h. After this time, one scratch per well was generated and images were taken on an EVOS imaging system at 4× for 8 h. The images were analyzed using the MRI Wound Healing Tool on ImageJ software (1.53k, U. S. National Institutes of Health, Bethesda, MD, USA).

#### 2.4.3. Cell Proliferation Assay

The proliferation of HMRECs was measured with the CyQUANT™ Cell Proliferation Assay kit (cat. #C35011) from Thermo Fisher Scientific Inc. (Waltham, MA, USA), following the manufacturer’s instructions. In a pretreated 96-well plate, 100 μL of the staining mixture was added and fluorescence was measured using the Synergy 2 microplate reader from BioTek Instruments, Inc. (Winooski, VT, USA), with an excitation wavelength of 485 nm and an emission wavelength of 530 nm.

### 2.5. Statistical Analysis

All measurements were performed at least in triplicate and plotted in box-and-whisker diagrams or bar graphs. Data were statistically evaluated using a *t*-test and one-way ANOVA to assess significant differences using GraphPad Prism 9.5.1 and were considered statistically significant with a *p*-value less than 0.05.

## 3. Results

### 3.1. RSV-NS Characterization

**Figure 2:**
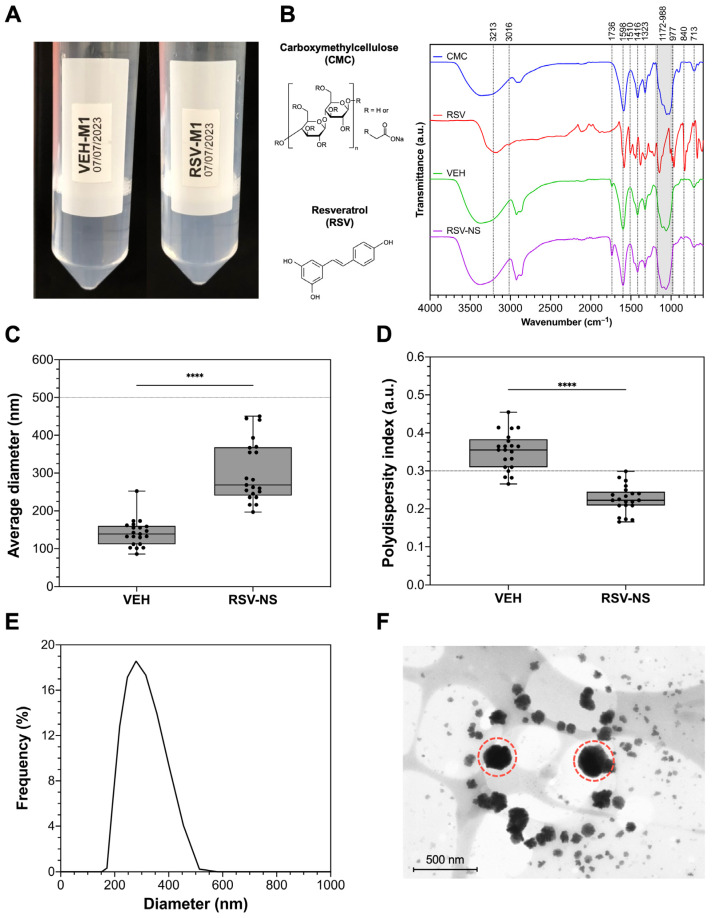
Physicochemical characterization of the formulation with resveratrol. (**A**) Appearance of the vehicle and the formulation with resveratrol after obtaining it. (**B**) Infrared spectra of carboxymethyl cellulose (CMC), resveratrol (RSV), vehicle (VEH), and the formulation with resveratrol (RSV-NS), along with their chemical structures, by Fourier transform infrared spectroscopy. Average particle diameter (**C**), average polydispersity index (**D**), and representative particle size distribution (**E**) of the formulation by dynamic light scattering; *n* = 21. Data were analyzed using an unpaired *t*-test. **** *p* < 0.0001. (**F**) Micrograph of the formulation using field emission scanning electron microscopy to visualize round-like particle morphology (red dashed circles). Magnification: 150,000×. Bar scale: 500 nm.

Figure 2 shows the physicochemical characteristics of VEH and RSV-NS, which, after 24 h, have a translucid appearance with no precipitants or phase divisions (Figure 2A). The FTIR spectra of RSV, CMC, VEH, and RSV-NS are presented in Figure 2B. A stretching band of the alkene group at 2903 cm^−1^ is shown in the CMC IR spectrum. Also, around 1598 cm^−1^ shows the characteristic band of the carboxylate ion COO–, and in the range of 1416–1323 cm^−1^, the bands of the hydroxyl group –OH, the skeletal vibration of the CO pyranose ring at 1054 cm^−1^, and the typical band of CH rocking at 713 cm^−1^ are visible [33,34].

A characteristic absorption band at 3213 cm^−1^ resulted from the free –OH stretching vibration for the RSV spectrum due to the alcohol group. There were bands at 3016 cm^−1^, indicating the stretching of the C–H group, and 1581 cm^−1^, which corresponded to the C=C functional groups, both related to the double-bond structure of the benzene ring from RSV. The presence of absorbance bands at 1510 and 1462 cm^−1^ reflects the vibrations of the benzene skeleton. The typical trans-olefinic form of RSV is demonstrated in the 988 and 967 cm^−1^ bands, where the bending vibration of C=C–H is observed [35,36,37,38]. The RSV-NS formulation can highlight the –OH stretch given by the band in the range of 3465–3257 cm^−1^ and the C–H stretch in the peak in the range of 2909–2858 cm^−1^. An aromatic compound, C=C, appears at 1736 cm^−1^; at 1598 cm^−1^, carbonyl group C=O, and in the range of 988–965 cm^−1^, a bending C=C–H band appear [33,39]. 

The particle size (PS) and polydispersity index (PDI) of VEH and RSV-NS are presented in Figure 2C,D, respectively, which are 141.1 ± 35.9 nm for VEH and 304.0 ± 81.21 nm for RSV-NS. Likewise, the average IPD for the VEH was 0.352 ± 0.049 and 0.225 ± 0.036 for RSV-NS. The particle distribution in Figure 2E indicates a uniform monomodal distribution and a size allowed within the range for ophthalmic applications. The micrograph of the RSV-NS is presented in Figure 2F, showing the particles’ spherical-like shape with diameters less than 500 nm and smooth edges.

### 3.2. Cell Cytotoxicity

MTT and CCK-8 assays use NADH/NADPH as cosubstrates, which are formed in the course of metabolic activity in pathways such as glycolysis, the pentose phosphate pathway, the tricarboxylic acid cycle, and mitochondrial oxidative phosphorylation [40,41]. Commonly, both assays are used to test the cytotoxicity of drugs in terms of cell viability and cell metabolic activity [42].

**Figure 3:**
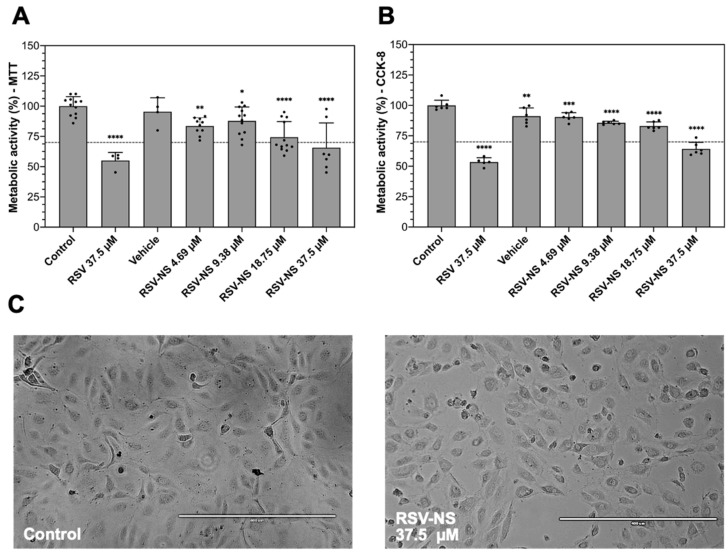
Percentage of metabolic activity through the reduction in salts of (**A**) 3-(4,5-dimethylthiazol-2-yl)-2,5-diphenyltetrazole (MTT) and (**B**) the Cell Counting Kit-8 (CCK-8) on human microvascular retinal endothelial cells (HMRECs) treated with free resveratrol (RSV), vehicle, and the resveratrol nanosuspension (RSV-NS) at different concentrations, compared to the unstimulated control; * *p* < 0.05, ** *p* < 0.01, *** *p* < 0.001, and **** *p* < 0.0001; one-way ANOVA with Fisher’s LSD post hoc tests; *n* ≥ 4 represents as mean ± standard deviation. (**C**) Comparison of the morphology of HMRECs treated for 24 h with RSV-NS at 37.5 μM. Scale bar: 400 μm.

Figure 3A presents the cell viability graph according to different RSV concentrations in RSV-NS, using MTT. It can be seen that the cells treated with free RSV at 37.5 µM show moderate cytotoxicity since the metabolic activity is 55.1 ± 6.65%. The nanosuspensions with concentrations higher than 18.75 µM have a cytotoxic effect on the cells. At 37.5 µM of RSV-NS, the metabolic activity is 65.73 ± 20.4%, denoting that the cytotoxic effect is moderately significant. On the other hand, the cells treated with the formulation at 18.75 µM have a viability of 74.34 ± 12.87%, demonstrating that the effect is slight. Cells treated with an RSV-NS concentration lower than 18.75 µM do not show a cytotoxic response.

To validate the results obtained by MTT reduction, CCK-8 containing another tetrazolium salt was used. CCK-8 uses a water-soluble tetrazolium salt that converts to orange formazan upon bioreduction in the presence of electrons; it has excellent stability, low toxicity, allows for prolonged incubation for up to 48 h, and has higher detection sensitivity than other tetrazolium salts, such as MTT, MTS, or XTT [43]. 

In Figure 3B, it is shown that cells treated with the RSV control at 37.5 µM present a metabolic activity of 53.45 ± 3.5%, compared to the control (100 ± 4.2%), indicating that it has a moderately cytotoxic effect. Nonetheless, the only concentration of RSV-NS that presents cytotoxic effects is 37.5 µM, with a metabolic activity of 64.24 ± 5.38%, considered as a slightly significant impact. For concentrations of 18.75, 9.38, and 4.69 µM, the metabolic activity values are 83.05 ± 3.36%, 85.65 ± 1.34%, and 90.44 ± 3.56%, respectively. There are no morphological changes between the control and the cells treated at 37.5 µM of the RSV-NS, as shown in Figure 3C.

### 3.3. Cell Migration and Proliferation

**Figure 4:**
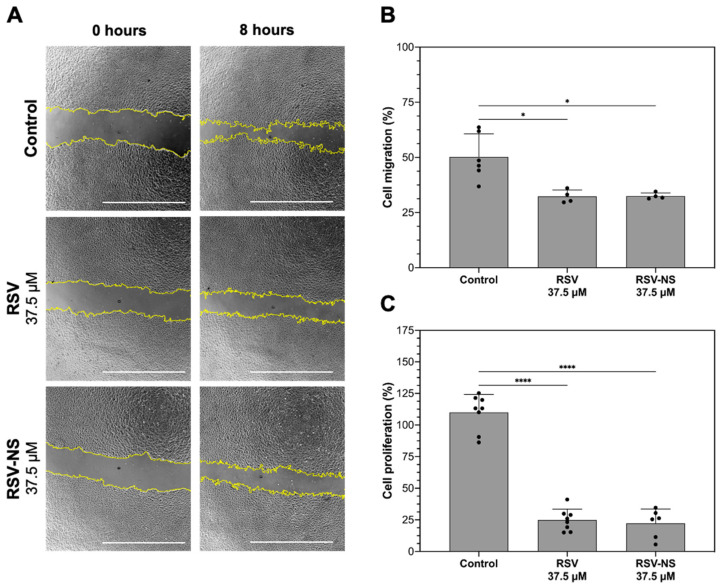
(**A**) Cell migration assay by wound closure for 8 h in human microvascular retinal endothelial cells (HMRECs) treated with free resveratrol (RSV) and the resveratrol nanosuspension (RSV-NS) at 37.5 μM for 24 h. The yellow lines indicate the wound’s initial and final areas. Scale bar: 1000 μm. (**B**) Percentage of cell migration at 8 h and (**C**) cell proliferation compared to the unstimulated control at 24 h; * *p* < 0.05 and **** *p* < 0.0001; one-way ANOVA with Fisher’s LSD post hoc tests; *n* ≥ 4 represents as mean ± standard deviation.

It was possible to determine if the RSV-NS had any effect according to the intrinsic migration of cells through the wound healing assay. Figure 4A,B illustrates the migration results for each treatment. It is shown that the cells treated with RSV-NS present a significant lower percentage of migration (32.48 ± 1.42%) in comparison to the unstimulated control (50.21 ± 10.49%) at 8 h. In addition, free RSV significantly reduces the migration (32.38 ± 2.94%) to the control. The RSV in the formulation does not present significant differences between free RSV. Secondly, RSV-NS significantly decreases cell proliferation on HMRECs at 37.5 µM (22.28 ± 11.33%) compared to the unstimulated control (109.9 ± 14.24%), with non-significant differences with free RSV at the same concentration (24.83 ± 8.71%), as shown in Figure 4C.

## 4. Discussion

Oxidative stress and inflammation promote the initiation and progression of DR, and there is evidence that anti-oxidative and anti-inflammatory phytochemicals, including RSV, can have therapeutic potential for this disorder [44]. However, RSV has certain limitations due to its chemical nature, such as photosensitivity, low bioavailability, and easy degradation [45,46]. Therefore, it is necessary to include it in a suitable vehicle for its delivery. Nanosuspensions have generated much attention and have become an important line of research in nanotechnology. They are colloidal systems containing nanometric drug molecules stabilized by surfactants/polymers [47] that have been widely used to improve the poor solubility and bioavailability of drugs, which increases their effectiveness [48]. 

In this research, we obtained an RSV-NS, where the drug particles were stabilized with two surfactants, T20 and S20, together with a cosolvent (GLC). Both T20 and S20 are nonionic surfactants with a hydrophilic–lipophilic balance of 16.7 and 8.6, respectively, with T20 being hydrophilic and S20 being hydrophobic, and have been used as solubilizing agents for a variety of substances, including fat-soluble vitamins and essential oils [49].

The drug nanoparticles were dispersed in an aqueous phase with CMC. This polymer has also been used as a stabilizer for nanosuspensions, and it is characterized by high chemical stability, non-toxicity, and biocompatibility [50]. The FTIR spectrum of the RSV-NS suggested that RSV was completely wrapped in CMC [51], by means that the amplitudes of the characteristic absorption peaks of RSV decreased dramatically, disappearing even [52], and this could indicate the formation of the drug nanoparticles that reduced the bending and stretching of the bonds in RSV due to the incorporation of resveratrol within the surfactants [38,52]. However, most of the CMC peaks were preserved in the nanoparticle, effectively indicating the dispersive effect of CMC in the formulation process.

Particle size is an essential characteristic to achieve optimal activity in controlled drug release since it not only affects drug solubility and dose uniformity, but can also affect the bioavailability of the active ingredient at the site of interest [53]. Previous research indicates that only 5% of the active ingredients can pass through the cornea and reach the intraocular tissue due to the rapid loss caused by high tear-fluid production or blinking [54], which can be optimized with the use of nanoformulations. According to the previous research, for ophthalmic applications in the posterior segment, it is ideal that the particle size does not exceed 500 nm so that the diffusion of the active ingredient through the various barriers of the eye is possible [55]. 

The nanosuspension obtained in this work had a particle size suitable for ophthalmic applications (304.0 ± 81.21 nm). In addition, it is reported that when drops dispersed in a nanosuspension are of the order of microns, the appearance is milky-like. Still, by decreasing the size of the drops to the order of nanometers, the appearance becomes translucent or transparent, which results in a significant marketing interest for this application [56]. The small size provides optical transparency, stability against sedimentation, and kinetic stability to the NS [56]. The PDI of the formulation was less than 0.3, indicating that it had a narrow size distribution and high homogeneity [57,58]. Also, with their spherical-like morphology, they are not expected to cause eye irritation, and we believe that they can have a longer pre-corneal retention time compared to formulations having larger particle sizes [59]. After obtaining the RSN-NS, the particle size, particle distribution, and PDI were considered adequate for this implementation, and it suggested a higher surface-to-volume ratio and faster dissolution of the drug, providing better stability, biodistribution, and penetration into the inner mucin layer of the tear film [23,60].

To evaluate the in vitro effects of the nanosuspension, the cell activity was measured as an indicator of cell damage or cytotoxicity in healthy HMRECs, based on the ISO 10993 standard of 2009, which considers that a reduction in cell viability by more than 30% is regarded as a cytotoxic effect [61]. In a certain way, when working with anti-inflammatory and antiangiogenic compounds, it is expected to see a reduction in cell proliferation, sufficient to reduce abnormal retinal neovascularization but without being severely cytotoxic, which can destabilize the cellular environment and cause collateral damage, especially in healthy cells [62]. Therefore, we considered that the nanosuspensions with a drug concentration of less than 37.5 were adequate for this application because their effect on this type of cell was severely cytotoxic. It can be highlighted that the higher the concentration of RSV in the formulation, the percentage of viability decreases. However, cell proliferation and migration were significantly reduced without being severely cytotoxic to the cells.

Other authors, such as Rokicki et al. [63], also reported this result and evaluated the in vitro influence of RSV on the proliferation, migration, and invasion of an immortalized murine endothelial cell line. RSV was shown to inhibit endothelial cell viability in the MTT (at 1, 10, and 50 µM) and AlamarBlue (at 50 µM) assays; however, the maximum concentration of 100 µM of resveratrol was cytotoxic for this cell line and was therefore not considered during subsequent experiments. Additionally, it was shown that RSV had an inhibitory effect on hyperglycemia-induced inflammation in retinal pigment epithelium cells (ARPE-19), where cell viability in a high-glucose medium (33 mM) decreased by increasing the RSV concentration from 1.25 to 10 µM. However, the viability of RSV-treated cells was not significantly different from the viability of the glucose control at average concentrations (5.5 mM) [64]. 

It is known that hyperglycemia related to diabetes can induce high concentrations of reactive oxygen species (ROS) through various mechanisms, to which the retinal cells are the most exposed to suffer from oxidative stress and lipid peroxidation, thanks to the fact that they are extremely rich in polyunsaturated lipid membranes and exhibit high oxygen consumption [65,66,67]. Lately, considerable attention has been paid to the use of RSV to counteract the adverse effects produced by oxidative stress, angiogenesis, and inflammation in diabetic retinopathy due to various studies that have shown its ability to control glucose metabolism, decrease insulin resistance, and prevent lipoprotein oxidation, apoptosis, and the inhibition of platelet aggregation [44,68,69,70]. It is necessary to study the mechanism of RSV in inflammation and angiogenesis, which are related to DR progression. Furthermore, it is essential to include studies of the antioxidant capacity of the nanosuspension and analyze the stability of the obtained dispersions as a function of time in future investigations. 

## 5. Conclusions

This research allowed us to obtain a nanosuspension of carboxymethylcellulose and resveratrol (RSV-NS) characterized by a translucent appearance, which did not present sedimentation for more than 24 h. This formulation has a particle size that meets the standards for ophthalmic applications of less than 500 nm, with a uniform particle distribution and a spherical morphology that can allow for a homogeneous release of RSV. The results show that RSV is loaded in the nanosuspension according to FTIR. The effects of this nanosuspension on HMRECs were analyzed under healthy conditions. The formulation had no severe cytotoxic effects at RSV concentrations less than 18.75 µM according to the MTT and CCK-8 assays, thus confirming that cell viability decreased at increasing RSV concentrations in the nanosuspension in the culture media. Likewise, proliferation and migration rate were significantly decreased by 87.6% and 17.7%, respectively, when the cells were treated with 37.5 µM of RSV-NS compared to the unstimulated control. In future experiments, this investigation can be further enriched by evaluating the nanosuspension in stressed cells to simulate in vitro conditions in DR, by testing the effect of RSV formulations in either increasing the glucose concentration in the media or by stimulation with inflammatory cytokines as compared to healthy cells. The in vitro evaluation of potential drugs indicates an important step for controlling this microvasculopathy. As an active ingredient that modulates inflammation, RSV shows promise in reducing ocular proinflammatory mediators. Thus, this nanosuspension can substantially contribute to future medical implementations and has a high potential to treat diabetic retinopathy.

## Data Availability

Data are contained within the article.
